# Study of summer microclimate and PM_2.5_ concentration in campus plant communities

**DOI:** 10.1038/s41598-024-52508-3

**Published:** 2024-02-09

**Authors:** Yuan Jiang, Congzhe Liu, Chenjie Wen, Yuelin Long

**Affiliations:** 1 College of Horticulture, Hunan Agricultural University, Changsha, 410128 People’s Republic of China; 2 College of Landscape Architecture, Nanjing Forestry University, Nanjing, 210037 People’s Republic of China; 3 College of Landscape Architecture and Art Design, Hunan Agricultural University, Changsha, 410128 People’s Republic of China

**Keywords:** Atmospheric science, Climate change, Urban ecology, Environmental impact

## Abstract

Understanding the influencing effect of meteorological factors and air pollutants in the campus plot and the relationship between them is an important topic in the planning and design of campus green space. The changes of pollutant concentrations and meteorological factors in campus green space have certain patterns and specific influencing factors. In this study, we selected four sample plots in Nanjing Forestry University as the research objects, and collected various environmental parameters of the four plots on July 25, 2022. The results showed that the main influences of meteorological factors are the type of the underlying surface of the site, the degree of plant canopy density and the shade coverage area of the building. These factors mainly have a great influence on the value of temperature and humidity. The comprehensive influencing factors can be concluded that the cooling and humidifying effect of the site is ranked as follows: forest > lawn > asphalt road > concrete Square. The main influencing factors of pollutants are: illumination, wind speed, temperature and relative humidity. Among them, illumination and temperature have a negative correlation with PM_2.5_, wind speed and relative humidity have a positive correlation with PM_2.5_. Our research shows that the adjustment of campus green space factors can reduce the concentration of pollutants by changing the meteorological factors.

## Introduction

Although urbanization has improved the living standards of urban residents, it has also produced a series of environmental problems, such as urban heat island effect, air pollution and ecological imbalance^1^. Among these problems, air pollution seriously affects the air quality of the city and the health level of the residents^2,3^. However,urban vegetation has become one of the primary means of alleviating and preventing urban air pollution, and it has a significant improvement effect on the small environment near green spaces^4,5^.

In the campus green space, the changes in meteorological factors (temperature, relative humidity, wind speed, radiation, diurnal cycle) also have a significant impact on human physical health^6^. Also, the factors in green space such as underlying surface, canopy density, building shadow coverage and so on, have a significant impact on meteorological indicators^7^. For example, sample plot underlays can radiate heat and help improve the microclimate^8^. The plant canopy density and the shade coverage produced by the building can reduce the air temperature, the length of the building shading time can improve the outdoor comfort of the plot, and the plant canopy density can reduce the air temperature and increase the air humidity^9^. Not only that, green space also indirectly affects the concentration of PM_2.5_ by affecting meteorological factors^10^. However, research on the impact of green spaces on meteorological factors mainly focuses on the analysis and research of large-scale green areas such as urban parks and urban forests, while there is less research on small-scale green space communities^11,12^. So studying the impact of campus green space communities on microclimate has important practical significance.

Ambient PM in Nanjing occurs from primary sources such as motor vehicles and coal combustion^13,14^. Owing to the differences in energy consumption and meteorological conditions among different seasons, there are daily and seasonal changes in the ambient PM concentration in Nanjing^15^. A previous study showed that the annual mean mass fractions of PM_2.5_ and PM_10_ in Nanjing exceeded the secondary standard limits of "Ambient Air Quality Standard" by 44% and 38%, respectively^16^. The concentrations of different particle size fractions varied significantly among seasons^17^. PM_2.5_ and PM_10_ concentrations in spring are 3.1 and 1.9 times lower than in winter, primarily because of intensive emissions from coal burning for domestic heating^18^. The daily peak concentrations of PM occur at 7:00‒8:00 and 19:00–20:00^19^. The reduction of inhalable particulate matter by the campus green space does not have the influence of traffic flow and morning peak on the concentration, but there are certain seasonal changes^20^.The Urban Green Space (an area of open land on which building is restricted) can improve air quality by blocking dust through interception and fixation of atmospheric dirt^10,21^. Studies on solving environmental pollution problems should include more than the management of pollution sources at present. Urban greening has become an effective way to alleviate the pressure of air pollution and therefore is regarded as an important quantitative indicator for evaluating the environmental benefits of green spaces^22^. Previous research showed that the concentration of PM with aerodynamic diameters ≤ 2.5 µm (PM_2.5_) at each research point presents a significant positive correlation with the relative humidity in the community. However, the correlation was insignificant regarding canopy closure, lawn coverage, and atmospheric pressure^23^. Plants can shield 36% of the particulate matter, and different plant communities can reduce the concentration of NO_2_ and SO_2_ by 10% to 30%^9,24^. In addition, the three-dimensional green mass of different plant communities in open spaces correlated significantly in a positive manner with pollutants^25^. The effect is also influenced by external environment factors, such as wind speed (WS) and direction^7,26^. Substantial differences were also observed for PM retained by different plant communities, such as arbors, shrubs, herbs, conifers, and broad-leaf deciduous and evergreen trees^8,27^.

As an important part of urban land, university campus is not an independent closed system, it plays an important ecological adjustment function and service function in the city^28^. With the proposal of the concept of green campus in 1996, campus construction began to develop in the direction of "low carbon, energy saving, and humanization", prompting people to pay more attention to campus green space^10^. The air quality in the campus, especially the concentration of PM and NO_2_ and other polluting gases, has an important impact on the health of students and teachers^29^. However, there are very few published studies on the campus environment, and due to regional differences, these studies are not fully applicable to the campus environment. Therefore, this paper selects the campus as the research area, and research on the PM_2.5_ reduction ability and microclimate regulation ability of green plant communities on the campus axis.

## Method

### Study area

**Figure 1:**
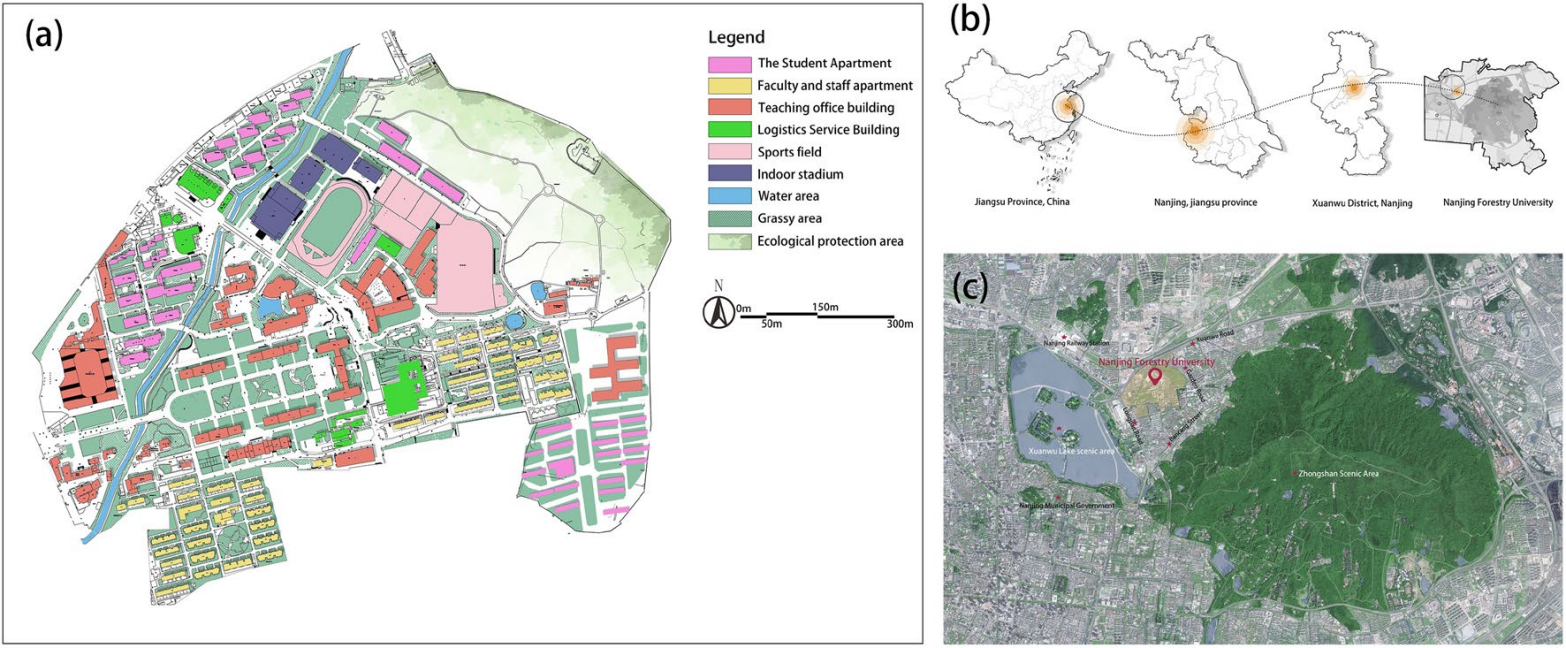
(**a**) Map of Campus land use plan; (**b**) Map of the location of campus; (**c**) Map of the investigated campus with surrounding areas.

The research is carried out on the central axis of the main campus of Nanjing Forestry University in Xuanwu District, Nanjing, Jiangsu Province (Fig. 1). Nanjing City was located in the lower reaches of the Yangtze River, in the eastern part of China, with latitude 31°14′–32°37′N and longitude 118°22′–119°14′E. The terrain of Nanjing is long from north to south and narrow from east to west, in a north–south direction; On the south is a geomorphic complex composed of topographic units such as low mountains, hills, valley plains, lakeside plains, and riverside land. Nanjing climate can be classified as northern subtropical humid, with four distinct seasons and abundant rainfall. The Nanjing Forestry University (Xinzhuang campus) we studied is adjacent to Zijin Mountain in the East and Xuanwu Lake in the West. There are many types of green space on unified campus axis.

### Plots description

**Figure 2:**
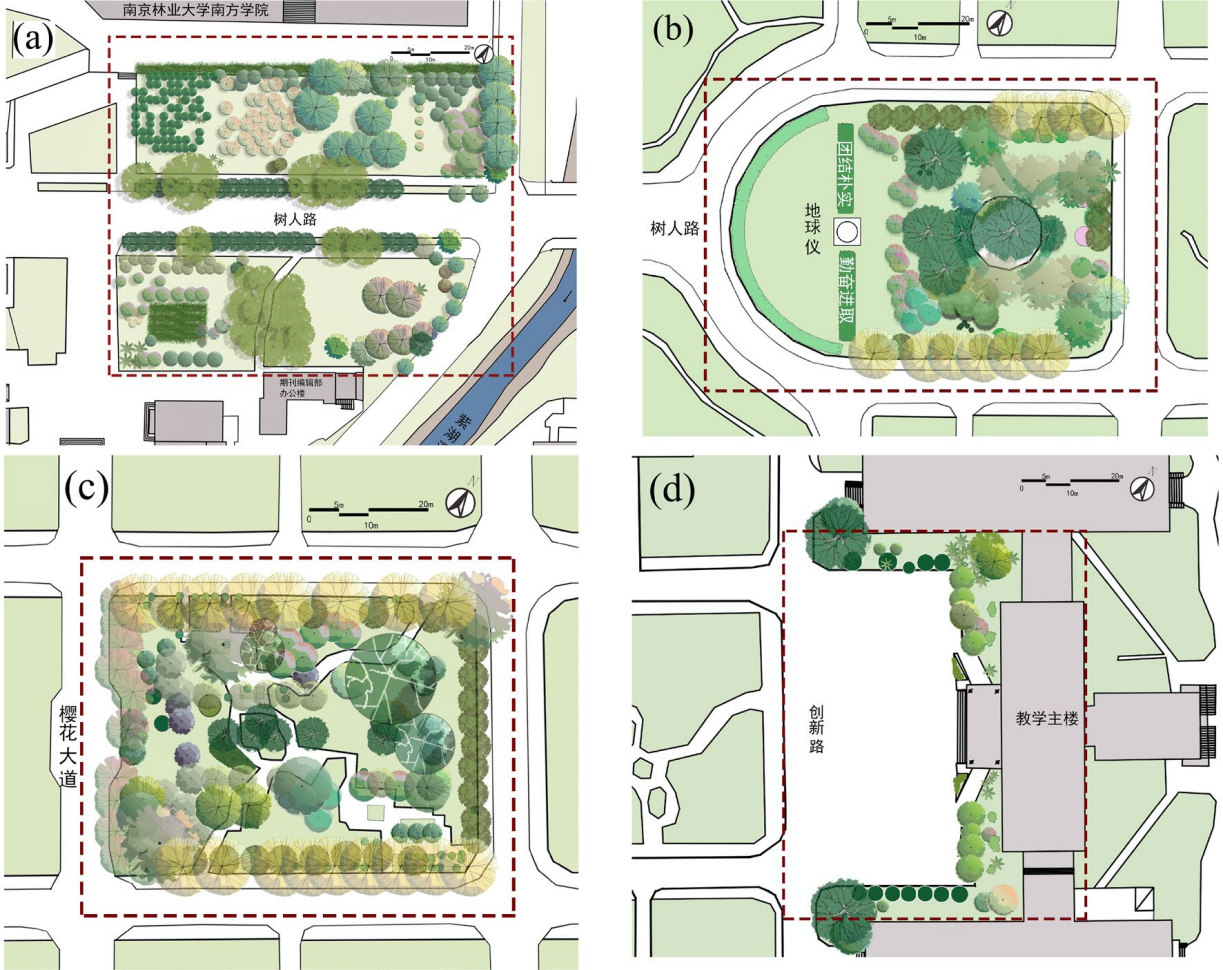
Plant distribution map of four plots on campus.

The four plots we sampled are located on the central axis near the west gate of Nanjing Forestry University (Fig. 2). The sample plots studied can be representative of the characteristics of various types of space on campus. The underlying surfaces of sample plot are asphalt road (plot a), grass land (plot b), bare soil (plot c), and concrete pavement (plot d) .

**Figure 3:**
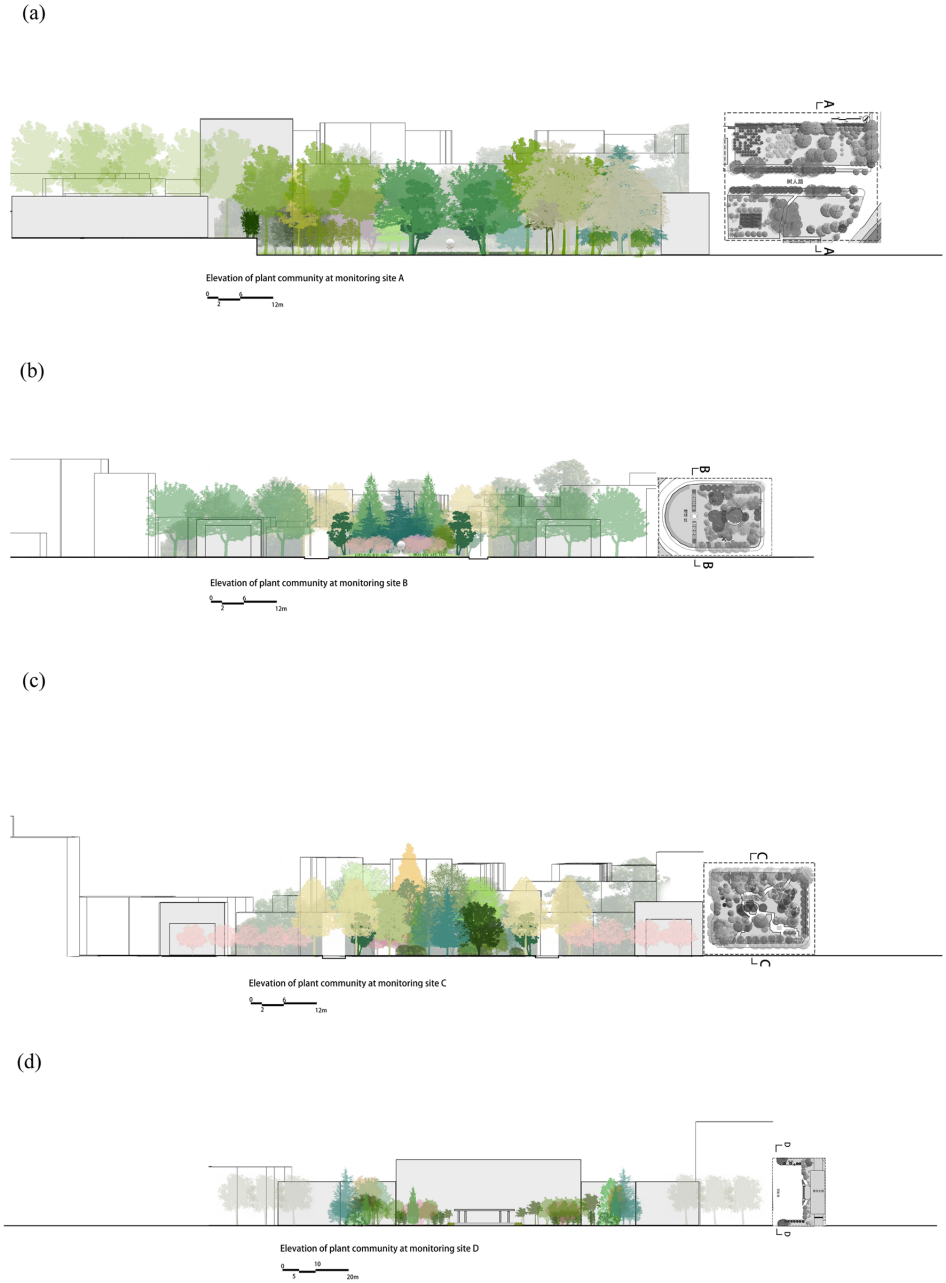
Elevations of the four plots (**a**) Elevations of plot 1 (**b**) Elevations of plot 2 (**c**) 6E5l65evations of plot 3 (**d**) Elevations of plot 4.

The vegetation information: Plot a is the roadside greenbelt, roadside was planted with 30–50 m of arbor (*Platanus acerifolia, Juniperus chinensis Roxb, Michelia platypetala*) with shrub (*Liriope platyphylla*) communities. Plot b is a semi-open green space, the west half is a lawn (*Buxus sinica* + *ineckia carnea*), and the east half is an green space with trees. Plot c is a pure forest green space with trees (*Quercus aliena, Sapium sebiferum, Cerasus yedoensis, Sabina chinensis ‘Kaizuca’, Sabina chinensis, Magnolia grandiflora*) and shrubs (*Fatsia japonica, Pittosporum tobira, Euonymus japonicus*). Plot d is a square without vegetation coverage (Fig. 3; Table 1).

**Table 1:** Plant community types at the sampling site.

Experimental plot	Plant species	Community type
Plot a	*Platanus acerifolia, Juniperus chinensis Roxb ,Michelia platypetala* + *Liriope platyphylla*	Arbor + grass
Plot b	*Buxus sinica* + *ineckia carnea*	Shrub + grass
Plot c	*Quercus aliena, Sapium sebiferum, Cerasus yedoensis, Sabina chinensis ‘Kaizuca’, Sabina chinensis, Magnolia grandiflora* + *Fatsia japonica, Pittosporum tobira, Euonymus japonicus*	Arbor + shrub
Plot d		square

### Monitoring network and sampling

The field measurement was carried out in the central axis green space of Nanjing Forestry University on July 25. The outdoor meteorological datas and pollutant datas were collected at the four sample plot 1, 2, 3, and 4. The experimental times for plot 1, 2, 3, and 4 as follow, 8–12 a.m., 15–19 p.m. As shown on the table, The geographic coordinates, Noise, Longitude and Latitude were also recorded, The device had a measurement range of 1–1000 μg/m^3^, a resolution of 1 μg/m^3^ on PM, and the error is ≤  ± 10 μg/m3. The working environment had a temperature range from − 40 to 80 °C, a resolution of 0.1 °C, and an error of ≤  ± 2 °C. The detector had a range of 0–100% Relative humidity (RH) with a resolution of 0.1% RH and an error of ≤  ± 2% RH. (Other meteorological indicators are shown in Table 2).

**Table 2:** The measuring range and accuracy of Lift Environmental detector.

Testing content	Measurement range	Resolution ratio	Precision	Testing content	Measuring range	Resolution ratio	Precision	
PM_2.5_	0–1000 μg/m^3^	1 μg/m^3^	± 10 μg/m^3^	PM_10_	0–1000 μg/m^3^	1 μg/m^3^	± 10 μg/m^3^	
NO_2_	0–100 ppm	0.01 ppm	≤ ± 3%	O_3_	0–100 ppm	0.01 ppm	≤ ± 3%	
Temperature	-40–80 ℃	0.1 ℃	± 2 ℃	Relative humidity	0–100%RH	0.1%RH	± 2%RH	
Wind speed	0–60 m/s	0.1 m/s	± 0.5 m/s	Atmospheric pressure	300–1200 hpa	1 pa	± 1.5 hpa	
Radiation	0–2000 μw/cm^2^	1	± 1 μw/cm^2^	Illumination	0–300 KLux	0.1 KLux	± 0.1 KLux	
Noise	30–120 dB	0.1 dB	< 2%					

### Data collection

**Figure 4:**
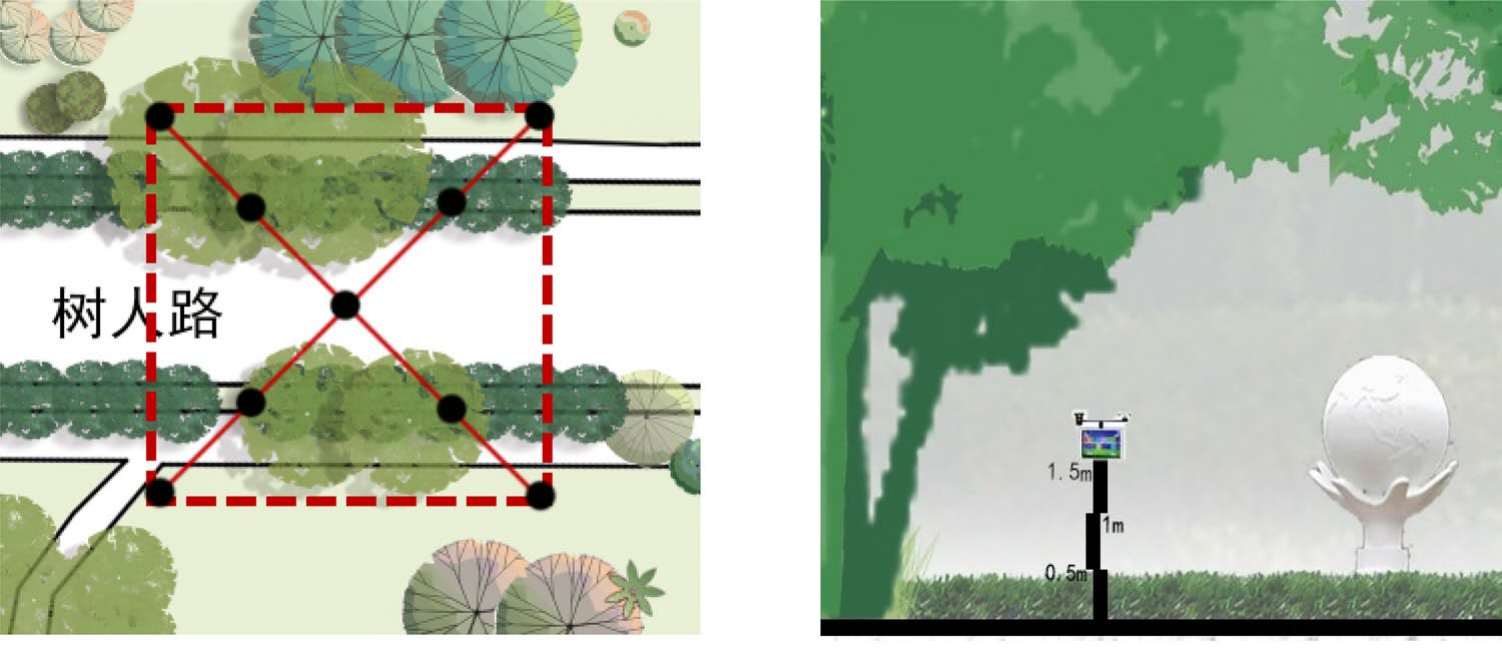
Distribution and height of research point in sample plot.

Concentrations of ambient PM_2.5_, PM_10_ ,NO_2_ and O_3_ measurements were came from the 4 sample plots inside campus. For the campus sampling site, the diagonal measurement is adopted. Considering the diagonal length of each site and the timeliness of measurement, each measurement point is divided at an interval of 5 m. The size of each sampling point is 20 * 20 m, and the observation points are 9 individual. Using the observation method of fixed-point positioning, the monitoring height is 1.5 m at the height of human breathing, and the measurement is carried out in the time range of 8:00–19:00. Five sets of data are collected at each measurement point, and the data is sampled every 30 s to ensure the data synchronization and comparability. Based on the previous collection of annual campus pollutant and meteorological data by the research group in the early stage. After data analysis, we found that the PM_2.5_ concentration in summer was the lowest, and the response effect to the environment was more significant than other seasons. And the data from July best represents the climate characteristics of summer. So data were collected in 2022.7.25. Ambient PM_2.5_, PM_10_, NO_2_ and O_3_ concentrations and Weather factor data (shown in Table 1) were collected in the weather with out wind (wind speed < 2 m/s) because in this situations the effect on the pollutant concentration could be negligible^30^ (Fig. 4).

### Data analysis

The canopy density was determined by estimating the percentage of sky blocked out by the vegetation using a foliage cover scale at five pointsin the plot (the centre of the grid and halfway between the centre and each of the plot corners); the average of estimates by two individuals was taken at each point, and the mean of all five points yielded the average vegetative cover for each monitoring site^31^.

## Result

### The influence of different factors on temperature and humidity

#### Time variation pattern of temperature and humidity

**Figure 5:**
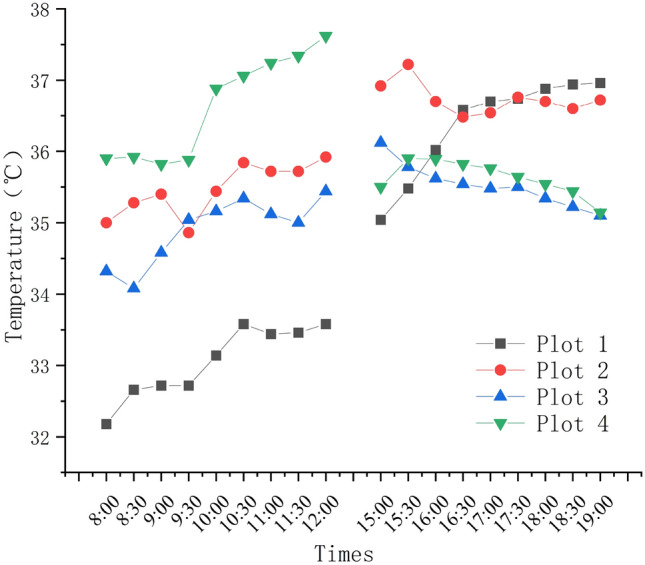
Daily changes of temperature at each test plot.

From the meteorological data obtained from the field collection, the pattern of the meteorological data change on the 4 plots of the campus can be analyzed. As the temperature data on Fig. 5, we can see that the temperature basically rise or drop with the detection time. From 8:00 to 12:00 a.m., the temperature gradually rises, and plor 4 has the highest temperature in the morning (37.62 °C). Thus, the lowest temperature appeared in the morning of plot 1 (32.18 °C). However, from 15:00 to 19:00 p.m. in the afternoon, the temperature in the sample plots basically showed a downward trend with time respectively. The lowest temperature appeared at plot 3 (35.10 °C), and the highest temperature appeared in the afternoon of plot 3 (37.22 °C). The temperature has the largest change in sample plot 1 and the smallest change in sample plot 3.

**Figure 6:**
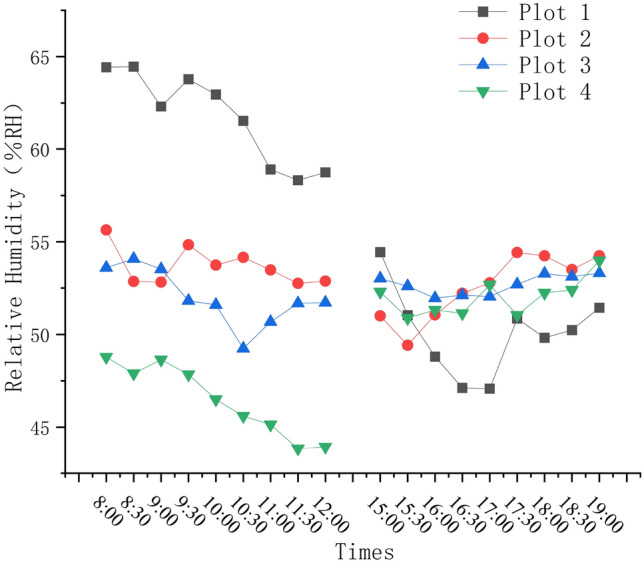
Daily changes of humidity at each test plot.

The humidity data showed a downward trend in the morning. The lowest point appeared at the sample plot 4 (43.93%RH), highest point appeared at the sample plot 1 (64.46%RH). In afternoon the humidity data showed an overall upward trend, while the humidity of plot 2, plot 3 and plot 4 remained basically unchanged, the lowest and highest humidity both appeared in plot 1. Summarizing the trend of humidity changes throughout the day, The relative humidity of plot 2 and plot 3 decreased slightly, and the relative humidity of the plot 1 and plot 4 has significant fluctuations (Fig. 6).

#### Illumination

**Figure.7:**
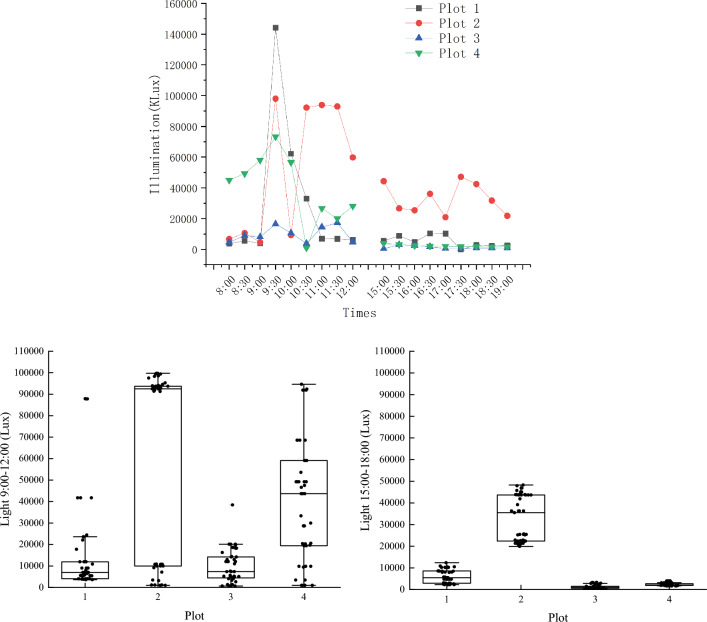
Daily changes of illuminations at each test point.

The changing trends of illumination are basically the same. In the morning, the illumination are higher than in the afternoon. The illumination at the plot 4 in the morning are at a high level and fluctuate greatly. Plot 3 has the lowest level of illumination and minimal fluctuations. The highest value of illumination throughout the day appears in the plot 1 (144,227.9Klux), The lowest value of illumination throughout the day appears in the plot 3 (457.7Klux), as shown on Fig. 7.

The illumination within the plot are closely related to the temperature and humidity, and the illumination and range within the plot are mainly effected by the canopy density of plants and the shadow coverage of buildings at different times (Table 3).

**Table 3:** Correlation analysis between light and temperature and humidity.

	Temperature	Humidity
Illumination	0.044**	− 0.337**

*Significant correlation at level 0.05 (two-tailed).

**Significant correlation at 0.01 level (two-Tailed).

##### Architectural Shadow

According to the changes of meteorological factors such as temperature and humidity at the site measurement points, this research analyzed the influence of building shadows. Compare the building shadow size of each sample plot in different time periods. Figure 4 shows the shadow situation of four points respectively. There was no rainfall or cloud cover on the measurement day, so the building shadow on July, 25, 2022 can be used for analysis.

**Figure 8:**
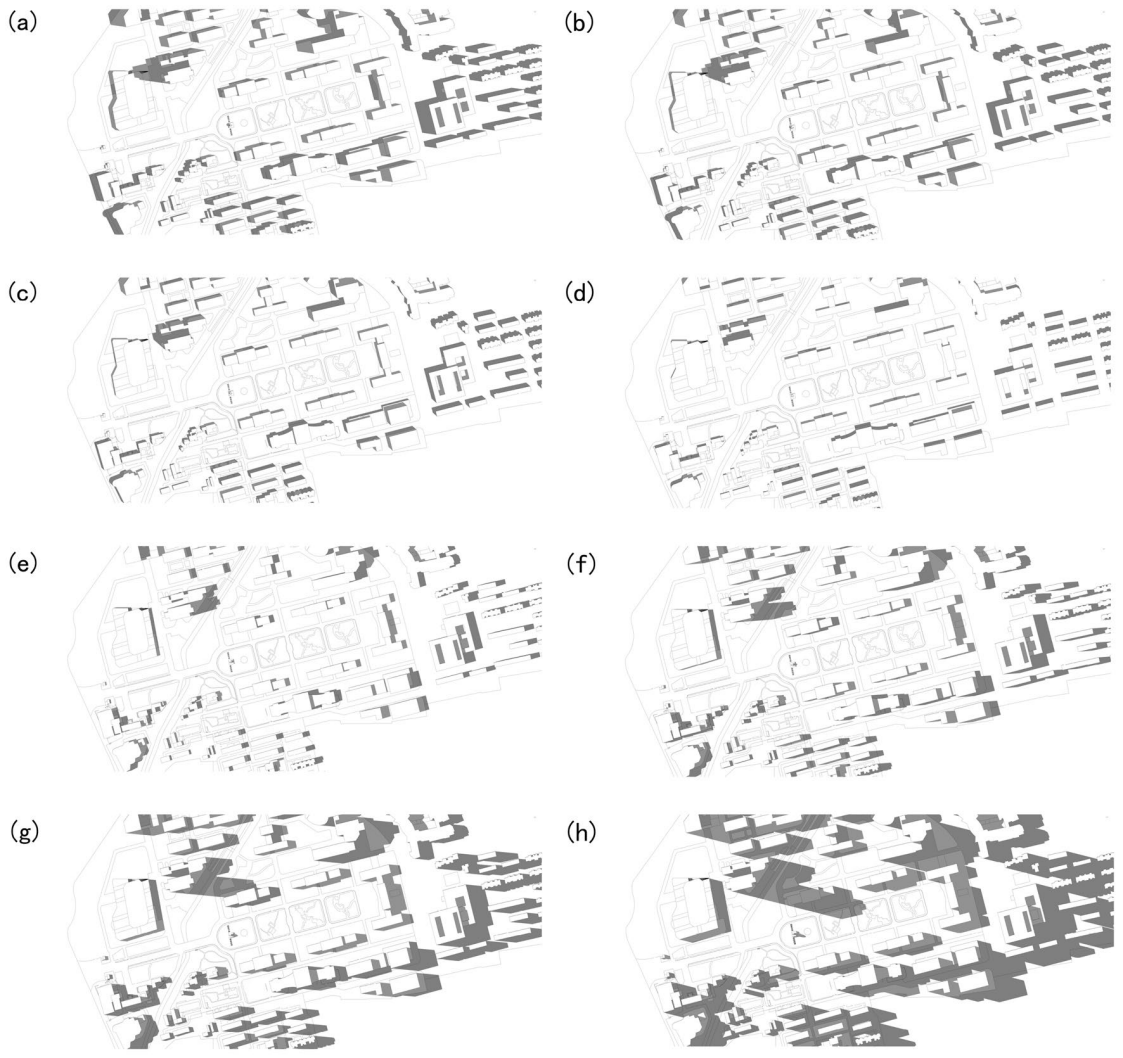
Shadow area of buildings on the central axis of Nanjing Forestry University (**a**) Shadow area of buildings on 9:00. (**b**) Shadow area of buildings on 10:00. (**c**) Shadow area of buildings on 11:00. (**d**) Shadow area of buildings on 12:00. (**e**) Shadow area of buildings on 15:00. (**f**) Shadow area of buildings on 16:00. (**g**) Shadow area of buildings on 17:00. (**h**) Shadow area of buildings on 18:00.

The sun rose at 5:08 and set at 19:09. The building-induced shadows lowered the air temperature at the shaded area. However, point 4 had a high average air temperature because the point was only covered by building-induced shadows at 19:20 and exposed to the sunshine in the remaining hours as displayed in Fig. 7. As shown in Fig. 7a-g, building shadows from 9:00 to 17:00 have little impact on plot, and in Fig. 7h on 18:00 o’clock building shadow covers plot 3, so the impact of building shadows on the sample plot is almost negligible (Fig. 8).

##### Canopy density

There are four types of plants communities in the four plots studied, Sample plot 1 is road green space; Sample plot 2 is lawn; Sample plot 3 is Pure Arbor Forest; Sample plot 4 is square, So the canopy density of the four plots is also different. The main area of the sample plot 1 is the internal roads of the campus and their road green space communities. On both sides of the sample plot 1 are street trees plus shrub community type, with a canopy density of 86%. The main area of the sample plot 2 is the lawn and green space community inside the campus, with trees on the east side and shrubs on the west side. There is no canopy coverage within the sample plot, and the canopy density is 25%. The main area of the sample plot 3 is the tree forest and green space community inside the campus, and the sample plot is fully covered by the tree vegetation community, with a canopy density of 94%. The main area of the sample plot 4 is the square in front of the teaching building inside the campus, and all the plots are square without vegetation cover, with a canopy closure of 0% (shown in Fig. 6).

**Figure 9:**
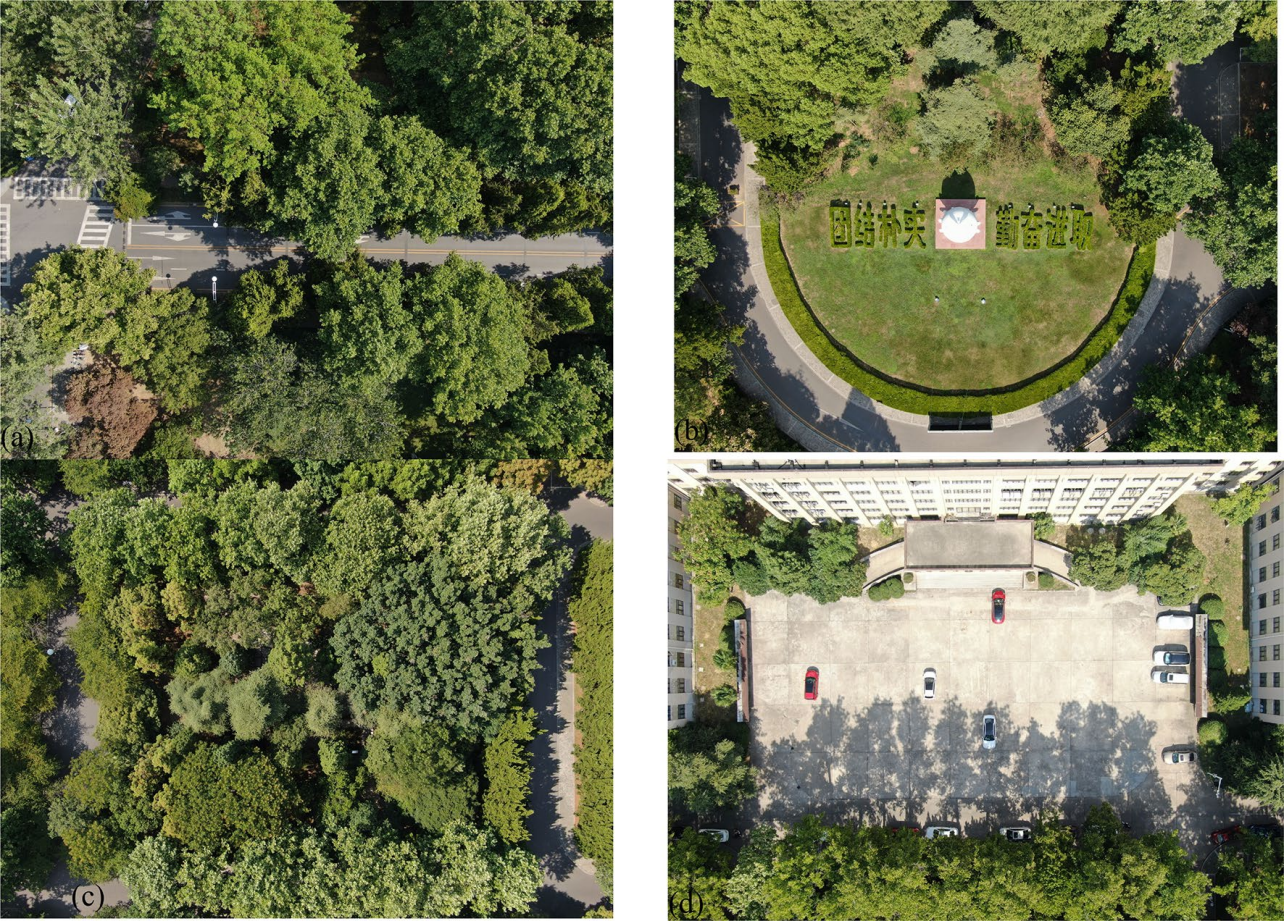
Canopy density of four sample plot.

The mean data shown on Table 2. The research has reported that sample plot 4 has the lowest canopy density 0, also was the plot with the highest temperature (36.15 ℃) and lowest humidity (49.20%) shown in Table 4. The underlying surfaces of the plot 1 and plot 4 samples are basically the same, only the changes in canopy density (0.76 and 0). By comparing plot 1 and 4, It was found that the temperature of sample plot 1 with high canopy density was lower than that of sample plot 4, but the humidity was higher than sample plot 4 (Fig. 9).

**Table 4:** Meteorological data changes under different underlying surface and canopy density.

Plot	Surface	Temperature (℃)	Relative Humidity (%)	canopy density	Notes
1	Asphalt pavement	34.62	55.93	0.86	Asphalt pavement underlying surface, surrounded by roadside trees
2	Lawn	36.11	53.11	0.25	The underlying surface of the lawn is surrounded by shrubs
3	Earth	35.20	52.33	0.94	Underlying surface of bare soil, surrounded by tall trees
4	Concrete pavement	36.15	49.20	0	Concrete pavement underlying surface, half enclosed by buildings and the other half enclosed by trees

#### Underlying surfaces

**Figure 10:**
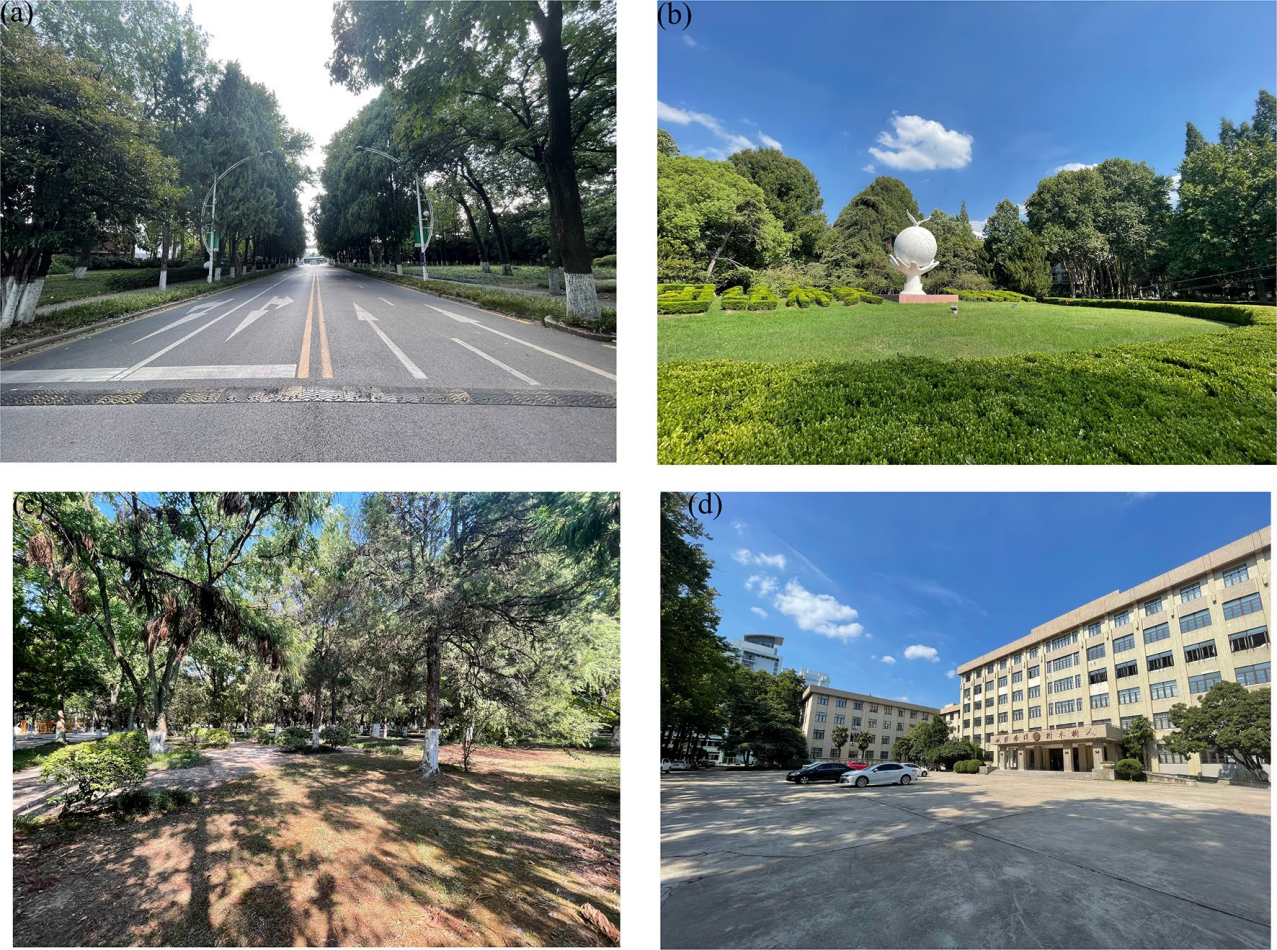
The underlying surface of the four sample plot.

There are four types of underlying surfaces in the four plots studied. Sample plot 1 is asphalt pavement; Sample plot 2 is lawn; Sample plot 3 is earth; Sample plot 4 is concrete pavement. (shown in Fig. 10).Thus, the canopy density of sample plot 2 and 4 were similar (0.25 and 0), and one underlying surface was lawn another was concrete pavement, and comparing the plot 2 and plot 4, It was found that the temperature of the underlying surface of the lawn and the temperature of the underlying surface of the concrete pavement basically do not changed much, but the relative humidity on lawn was rised (shown in Table 4).

### The influence of different influencing factors on PM_2.5_ concentration in campus greenery

#### Time and different plot Changes of PM_2.5_ concentration

**Figure 11:**
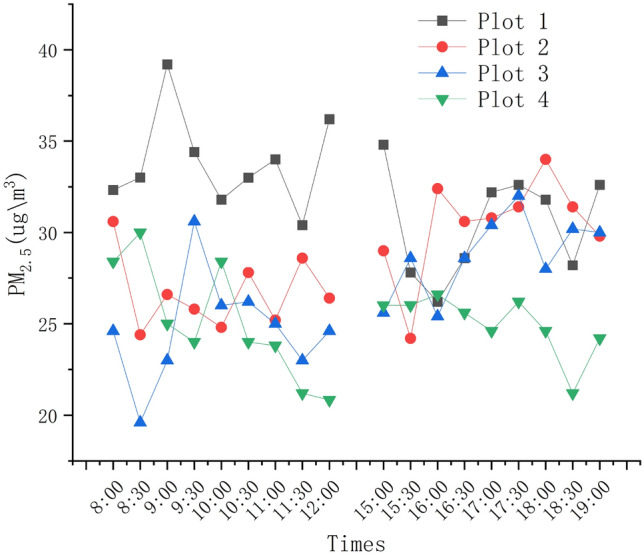
Daily changes of temperature at each test plot.

The diurnal variation pattern of PM_2.5_ concentration in summer (Fig. 11) shows an overall pattern of low in the morning and high in the afternoon, but the variation pattern of PM_2.5_ concentration in the Plot 1was opposite. The highest point of PM_2.5_ concentration in the morning occurs between 8:30 to 9:30, followed by a slow decline from 9:30 to 12:00, reaching its lowest value from 11:30 to 12:00, which is also the lowest PM_2.5_ concentration of the day. The lowest point of PM_2.5_ concentration in the afternoon occurs from 15:30 to 16:00, and the highest point occurs from 18:00 to 19:00, which is also the highest value of PM_2.5_ concentration in a day.

**Figure 12:**
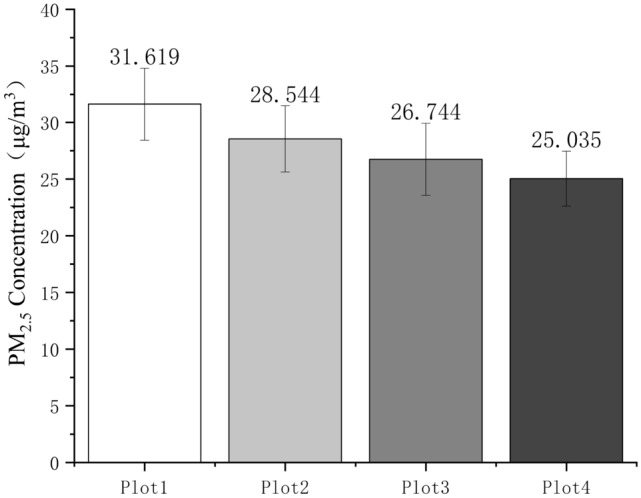
Differences in PM_2.5_ concentration at four plots.

The results of the changes in PM_2.5_ concentration at the four experimental plots in Fig. 12 indicate that. The experimental sample plot with the highest pollutant concentration is plot 1, and the lowest sample point is plot 4, with PM_2.5_ at four sample plots. The difference in PM_2.5_ concentrations is significant.

#### The influence of environmental factors on PM_2.5_ concentration

In the comparison of the correlations between 7 meteorological factors and the PM_2.5_ concentration, it is not difficult to conclude that the correlation between wind speed, Radiation, temperature and humidity and the PM_2.5_ concentration is relatively high, while the correlation between radiation and PM_2.5_ concentration is relatively high, Among the relations between them, PM_2.5_ concentration have strong negative correlation with temperature and radiation, while have strong positive correlation with relative humidity. However, there was no strong correlation between other meteorological factors and PM_2.5_ concentration (Table 5).

**Table 5:** Correlation between PM_2.5_ and meteorological environmental factors.

	Temperature (℃)	Relative humidity (%)	Wind speed (m/s)	Atmospheric pressure (pa)	Radiation (µw/cm^2^)	Illumination (Lux)	Noise (DB)
PM_2.5_ (µg/m^3^)	− 0.280**	0.476**	0.355**	− 0.035	− 0.140**	− 0.052	0.122*

*Significant correlation at level 0.05 (two-tailed)

**Significant correlation at 0.01 level (two-tailed).

## Discussion

### Analysis of temperature and humidity in campus green space communities

The influence of the underlying surface of the sample plot on the meteorological factors mainly lies in temperature and humidity. Because plants have transpiration, which is a process of absorbing surrounding heath, gas and releasing water vapor^32^. The temperature radiation of the impermeable hard underlying surface is greater than that of the green space, that is, the air temperature is higher than that of the green space^8^. The cooling order of different underlying surfaces is building < pavement < grass < forest. However, the influence of the underlying surface on humidity mainly lies in the degree of water permeability of the underlying surface and the degree of water evaporation on the underlying surface^33^.). The lower the humidity, the humidification order of different underlying surfaces is: building < pavement < grass < woodland.

The effects of canopy density and building shadows on the plots are mainly radiation, light intensity, cooling and humidification. Studies have shown that the canopy density begins to have a certain cooling and humidification effect in green space from 10 to 31%. The cooling and humidification effect is significant when the canopy density exceeds 44%^34^. When the canopy density exceeds 67%, the cooling and humidification effect is significant and tends to be stable^35^. The cooling and humidifying effect of high canopy density mainly utilizes a shading effect of plant leaves, thereby reducing solar radiation, lowering temperature and increasing understory humidity^36^. For building shading, the longer the site is shaded by buildings, the lower the temperature will be.

In this study, due to the difference in record time, the temperature difference can not represent the full cooling capacity. But the meteorological data in the afternoon is relatively stable, which can be used as a reference for data comparison of different influencing factors. we can compare different underlying surfaces with the same canopy density. Both the plot 2 and the plot 4 have lower canopy density. However, since the cooling and humidifying effect of the plot 2 is better than that of the plot 4, it can be concluded that the grass underlay The cooling and humidifying effect of the surface is better than that of the concrete underlying. The order of cooling and humidifying the underground cushion surface for campus use is Lawn > Bare Soil > Asphalt > Concrete. Plot 1 and plot 4 have similar types of underlying surfaces, and the cooling effect of plot 1 is better than that of plot 4, so it can be concluded that the cooling and humidifying effect of green space with high canopy density is better than that low canopy density one. Based on the previous research, it can be concluded that the ranking of cooling and humidifying of campus plant communities effects is: Forest Land > Street tree > Lawn > Square. Therefore, the green space with high plant canopy density as the underlying surface has the best cooling and humidifying effect.

### Analysis of PM_2.5_ concentration in campus green space communities

The daily variation of PM_2.5_ concentration also proves that the concentration of PM_2.5_ is highly correlated with the temperature and humidity of the environment^37^. The temperature and humidity change greatly in the morning, so the PM_2.5_ concentration has big change. With the temperature increased with time, the PM_2.5_ concentration becomes lower. In the afternoon, due to the insignificant changes in temperature and humidity, the concentrations of PM_2.5_ was still in a downward trend^38^.). It can be speculated that the PM_2.5_ concentration may also have a more significant correlation with other meteorological factors, such as wind speed, radiation and so on.

In the campus green space, the relationship between PM_2.5_ concentrations and meteorological factors is mainly related to temperature, humidity, radiation and wind speed^39^. Wind speed was significantly correlated with PM_2.5_. This can be related to the location of the pollution source in the campus^40^. The main pollution source in the campus is particulate matter, and the concentration of other pollutants is not high, so the concentration of PM easily changed with the change of wind speed^41^.).

The relationship between PM_2.5_ concentrations and temperature with humidity was the most significant^42^.). With the increase of temperature, the concentration of PM_2.5_ has decreased, Thus with the increase of humidity, the concentration of PM_2.5_ has increased. Because, when the humidity in the air is high, PM_2.5_ is adsorbed by water vapor, causing more particulate matter to condense and accumulate in high humidity environments, making it difficult to diffuse, so in an environment with high humidity, so the concentration of PM_2. 5_ were high^43^. There is a negative correlation between PM_2.5_ and temperature, mainly because the high temperature environment has caused an increase in the temperature difference between the inside and outside of the plants community, thereby increasing the air flow rate inside and outside the community, makes the PM_2.5_ concentrations in plants community easy to diffuse^44^.

## Conclusion

In this study, we show the changes in temperature, humidity, PM_2.5_ concentrations and their influencing factors at four plots along the central axis of Nanjing Forestry University. The results show that the underlying surface, canopy density and building shadow have a greater impact on the temperature and humidity of the campus plots. The relationship with cooling and humidification is as follows: The cooling and humidifying sequence is: forest > lawn > asphalt road > concrete Square.

The influence of meteorological factors on the concentration of PM_2.5_ is mainly reflected in several meteorological factors such as temperature and humidity, radiation and wind speed.

Wind speed and Humidity was mainly positively correlated with PM_2.5_, Radiation (Illumination) and Temperature was mainly negatively correlated with PM_2.5_, and pollutants were roughly negatively correlated with temperature and negatively correlated with humidity. Research shows that the adjustment of campus green space (plants communities, underlying surface, Surrounding environment) an reduce the concentration of PM_2.5_ by changing the meteorological factors (temperature, humidity, windspeed, illumination). These research findings are beneficial to the planning and design of campus green space in the future, helping to regulate the microclimate of campus green space and reduce air pollution on campus.

## Data Availability

The datasets used and analysed during the current study available from the corresponding author on reasonable request.
