# Author Correction: Study of summer microclimate and PM_2.5_ concentration in campus plant communities

**DOI:** 10.1038/s41598-024-58859-1

**Published:** 2024-04-09

**Authors:** Yuan Jiang, Congzhe Liu, Chenjie Wen, Yuelin Long

**Affiliations:** 1 College of Horticulture, Hunan Agricultural University, Changsha, 410128 People’s Republic of China; 2 College of Landscape Architecture, Nanjing Forestry University, Nanjing, 210037 People’s Republic of China; 3 College of Landscape Architecture and Art Design, Hunan Agricultural University, Changsha, 410128 People’s Republic of China

Correction to: *Scientific Reports* 10.1038/s41598-024-52508-3, published online 09 February 2024

In the original version of this Article Yuelin Long was incorrectly affiliated with ‘College of Landscape Architecture, Nanjing Forestry University, Nanjing 210037, People’s Republic of China’. The correct affiliations are listed below.

College of Horticulture, Hunan Agricultural University, Changsha, 410128, People’s Republic of China

College of Landscape Architecture and Art Design, Hunan Agricultural University, Changsha, 410128, People’s Republic of China

The original Article has been corrected.

